# Rheological and biological properties of a hydrogel support for cells intended for intervertebral disc repair

**DOI:** 10.1186/1471-2474-13-54

**Published:** 2012-04-10

**Authors:** Karin Benz, Claudia Stippich, Claudia Osswald, Christoph Gaissmaier, Nicolas Lembert, Andreas Badke, Eric Steck, Wilhelm K Aicher, Juergen A Mollenhauer

**Affiliations:** 1 NMI Natural and Medical Sciences Institute at the University of Tuebingen, Reutlingen, Germany; 2 TETEC AG, Reutlingen, Germany; 3 NMI TT GmbH, Reutlingen, Germany; 4 BG-Trauma Center, Tuebingen, Germany; 5 Research Center for Experimental Orthopaedics, Orthopaedic University Hospital, Heidelberg, Germany; 6 Centre for Medical Research (ZMF), Dept. Orthop. Surg., University Hospital, Tuebingen, Germany; 7 Department of Biochemistry, Rush University Medical Center, Chicago, IL, USA

## Abstract

**Background:**

Cell-based approaches towards restoration of prolapsed or degenerated intervertebral discs are hampered by a lack of measures for safe administration and placement of cell suspensions within a treated disc. In order to overcome these risks, a serum albumin-based hydrogel has been developed that polymerizes after injection and anchors the administered cell suspension within the tissue.

**Methods:**

A hydrogel composed of chemically activated albumin crosslinked by polyethylene glycol spacers was produced. The visco-elastic gel properties were determined by rheological measurement. Human intervertebral disc cells were cultured *in vitro *and *in vivo *in the hydrogel and their phenotype was tested by reverse-transcriptase polymerase chain reaction. Matrix production and deposition was monitored by immuno-histology and by biochemical analysis of collagen and glycosaminoglycan deposition. Species specific *in situ *hybridization was performed to discriminate between cells of human and murine origin in xenotransplants.

**Results:**

The reproducibility of the gel formation process could be demonstrated. The visco-elastic properties were not influenced by storage of gel components. *In vitro *and *in vivo *(subcutaneous implants in mice) evidence is presented for cellular differentiation and matrix deposition within the hydrogel for human intervertebral disc cells even for donor cells that have been expanded in primary monolayer culture, stored in liquid nitrogen and re-activated in secondary monolayer culture. Upon injection into the animals, gels formed spheres that lasted for the duration of the experiments (14 days). The expression of cartilage- and disc-specific mRNAs was maintained in hydrogels *in vitro *and *in vivo*, demonstrating the maintenance of a stable specific cellular phenotype, compared to monolayer cells. Significantly higher levels of hyaluronan synthase isozymes-2 and -3 mRNA suggest cell functionalities towards those needed for the support of the regeneration of the intervertebral disc. Moreover, mouse implanted hydrogels accumulated 5 times more glycosaminoglycans and 50 times more collagen than the *in vitro *cultured gels, the latter instead releasing equivalent quantities of glycosaminoglycans and collagen into the culture medium. Matrix deposition could be specified by immunohistology for collagen types I and II, and aggrecan and was found only in areas where predominantly cells of human origin were detected by species specific *in situ *hybridization.

**Conclusions:**

The data demonstrate that the hydrogels form stable implants capable to contain a specifically functional cell population within a physiological environment.

## Background

Biological regeneration of degenerated or traumatically damaged intervertebral discs is a desirable goal, and in the meantime an experimental focus of regenerative medicine. In recent time, the re-injection into the damaged disc of autologous disc cells harvested from prolapses has been even performed in clinical trials [1,2]. While data from such trials are emerging and suggest at least partial clinical success, the procedure is technically not satisfying yet. The injection of a liquid suspension of cells into the disc tissue is not safe enough without any measures to warrant the cells to remain within the disc and not be ejected during initial loading of the disc upon patient motion. Beyond this safety aspect, the introduction of fresh cells may not deliver success without simultaneously restoring the nutritional requirements and the moderation or prevention of inflammatory insults.

This present situation led several research groups to consider hydrogels as a means to anchor cells *in situ*, with the specific advantage that hydrogels might improve the nutritional situation of a disc with respect to its water content and the additional mechanical benefits of rebuilding disc height and resistance to compression. Two main strategies are imaged in literature: the surgical implantation of a preformed implant (derived from cell-free applications of hydrogels), and the injection of *in situ *polymerizing formulations. The present situation is reviewed in [3,4]. The structural basis for cell-supplemented hydrogels span across a remarkably long list of molecular elements, including mammalian components such as gelatin, chondroitin sulfate, hyaluronan, collagen, plant polymers such as alginate, and synthetic polymers such as polyethylene glycol, poly glycolic acid, and polylactic acid or synthetic peptides. The crosslinking is achieved by redox reactions (thiols), condensations (polyacrylates), or complex formation (alginate, peptides). Some of those reactions are designed to exclude cellular components from untoward inclusion into the reaction cascade, thus avoiding toxic interactions; others are balanced to minimize reactions that include cell surface components, without being strictly selective. Cell survival has been reported to be good to excellent, with most of the reports initially relying on *in vitro *observations. From those, a limited number has already been applied in animal experiments, either in ectopic or intradiscal administrations, with so far none reported to generate local or systemic adverse reactions.

Within this broader context, we also developed a hydrogel to be injected as a fluid and to polymerize *in situ*. The hydrogel is based on maleinimide-activated human serum albumin and cross-links chemically by dithiol-polyethylene glycol after mixing in a dual chamber syringe system equipped with an interrupted-helical mixing chamber. The hydrogel is enhanced with hyaluronan (HA) and adjusted to a visco-elastic stage that allows the gel to creep rather than build up significant local pressure, thus intended to anchor cells within the defect compartment but not to re-build disc volume. The hydrogel displays anti-angiogenic properties in cell culture and in chorioallantois membrane tests [5]. Those properties are considered to be key elements for disc regeneration since recently published data underscore the negative effects of capillary ingrowth into inflamed disc tissues [6-9]. The high molecular weight crosslinked HA (Visiol^®^, TBS Chemedica, Munich) itself is passively interwoven with the albumin polymer since it does not participate in the polymerization reaction. HA is included in the recipe because of its well-described chondro-protective, anti-inflammatory and biomechanical benefits (for review, see [10]). Numerous other groups are therefore currently also considering to apply HA in tissue engineering of cartilaginous implants, alone or in combination with other materials, and in various stages of technical development [11-23].

Here we present first experimental evidence for the hydrogel to be a suitable carrier for anchoring of disc cells in an ectopic animal model. The hydrogel stabilizes a chondrogenic phenotype of the cells, and facilitates deposition of a cartilagenous extracellular matrix.

## Methods

### Animals

Subcutaneous implantation of the human preparation was performed in a double-homozygous severe combined immunodeficient (SCID) hairless outbred model (Crl:SHO-Prkdc^scid^Hr^hr ^mice; Charles River Laboratories; Sulzberg, Germany). The animal experiment was approved by the Committee on Animal Care of the State of Baden-Wuerttemberg (Germany).

### Hydrogel preparation

Activated human maleolyl-albumin, hyaluronan and cell culture medium without supplements and without serum were used as basis for the albumin gel. Cross-linking was achieved through a specific thio-polyethylene glycol (SH-PEG, Rapp Polymere GmbH; Tuebingen, Germany) spacer via Michael reaction of a maleolyl-group to a SH-group, as described before [24] (pending patents number: PCT/EP2008/005643, DPMA 10 2008 008 071.3).

For the production of maleolyl-albumin, 250 mg of human serum albumin (Sigma-Aldrich; Steinheim, Germany) were dissolved in 5 ml 1 M sodium borate (Sigma-Aldrich). Afterwards 75 μl 260 mM maleolyl-β-alanine in PBS/sodium borate (1:1) were added and incubated for 90 min at room temperature. 106 mg 3-maleimidopropionic acid N-hydroxysuccinimide ester (Obiter Research; Urbana, USA) were dissolved in 950 μl dimethylformamide. 500 μl of this solution were added to the albumin solution and incubated for another 60 min. at room temperature. Afterwards 500 μl 3 M sodium acetate (Sigma-Aldrich) were added and dialysed against phosphate buffered saline (PBS) for 1 h on ice three times. The dialysate was concentrated to 3.5 ml by ultrafiltration (YM-3 membrane, Millipore; Billerica, USA) and stored at -80°C.

To prepare 2 ml of hydrogel solution, 140 μl maleolyl-albumin (43 mM), 1,060 μl cell culture medium and 400 μl high molecular weight hyaluronan (20 mg/ml, Visiol; TRB Chemedica AG, Munich, Germany) were mixed and incubated for 5 min. at room temperature. The remaining 400 μl were reserved for the cell suspension.

The crosslinker solution comprised of 500 μl SH-PEG (10,000 g/mol, 15 mM SH-groups) in 0.1 mM HCl, to achieve a 1:1 ratio of maleolyl-groups to SH-groups when being mixed with the hydrogel/disc cell solution. The gel mixtures were submitted to a DIN/ISO 10993-4, -5, 10, -11 biocompatibility testing (including gels prepared from human albumin and of the test species mouse, rabbit, rat, and guinea pig) by BIOSERV Analytik & Medizinprodukte GmbH (Rostock, Germany) and passed successfully.

### Rheological measurement of the visco-elastic properties of the hydrogel and effect of storage of gel components on the gelation process

All gel experiments were performed using a dual chamber syringe system, with a 2-ml chamber filled with the human disc cells suspended in culture medium plus high molecular weight hyaluronan and the polymerization agent maleimide-activated albumin, the other chamber filled with the chemical crosslinker SH-PEG, as described before [5,24].

To examine the visco-elastic properties of the hydrogels and the effect of storage of gel components on the gelation process rheological measurements were performed with a Kinexus Pro rheometer (Malvern Instruments GmbH; Herrenberg, Germany). Per analysis day the instrument performance was checked before the measurement procedure using calibration oil as a standard (PRA Coating Technology; Hampton, UK). The two gel components were mixed and injected onto the bottom plate of the rheometer using a dual-chamber applicator syringe as described in [24] and in the introduction. After automatically adjusting the upper plate (1 Pa, plane configuration, diameter 20 mm, spacing 0.6 mm) a ring envelope was mounted around the piston to prevent water evaporation during the experiment. The measurements started approximately 30 sec. after gel mixture. The experimental conditions for detection of gel formation were: temperature 37°C, shear strain 0.05%, shear stress 1 Pa, oscillation frequency 1 Hz. The gelation process was monitored for a period of 20 min. The electronic system continuously detected and stored the storage modulus (G' in Pa) and the loss modulus (G'' in Pa) with a frequency of 1 Hz. The pressure of the upper plate on the gel was set to 1 Pa and was automatically controlled during the process of gelation.

### Human tissue harvest

For the SCID mouse experiment human intervertebral disc (IVD) tissues were harvested from six patients during spinal fusion surgery. The donors were between 18 and 61 years old, two of them were female and four were male. For the *in vitro *experiment, the IVD tissues were harvested from 2 patients undergoing nucleotomie after disc herniation (male, 37 years; female, 32 years) and 2 patients undergoing spinal fusion surgery (male, 46 years; female, 46 years).

Due to the nature of the surgical procedures, a clear distinction between annulus and nucleus pulposus tissue could not always be made. Therefore, we aimed to collect unspecified IVD cells rather than distinct populations. All human tissues were obtained from the BG Trauma Center in Tuebingen (Germany). The studies were approved by the local ethics committee and informed consent was obtained from all individuals participating in the study. The sterile tissue was placed into culture medium and instantly transported to the tissue culture facility.

### Cell culture

#### Human IVD cell isolation and culture

Tissue samples were washed twice in PBS (BioWhittaker; Verviers, Belgium) and then minced by scalpels. Extracellular matrix was enzymatically degraded overnight by incubation in DMEM high glucose medium (Invitrogen; Karlsruhe, Germany) containing 0.375 mg/ml collagenase B (Roche; Mannheim, Germany), 0.05 mg/ml hyaluronidase (Serva; Heidelberg, Germany) and 5% human serum at 37°C. Isolated disc cells were filtered through a 100-μm cell strainer to remove undigested tissue fragments and extracellular matrix debris. After centrifugation, the cells were resuspended in DMEM/Ham's -F12 cell culture medium supplemented with 5% human serum (Centre for Clinical Transfusion Medicine, University of Tuebingen, Germany), 150 μM ascorbic acid-2-phosphate, 100 U/ml penicillin, 100 μg/ml streptomycin and plated in 75 cm^2 ^cell culture flasks at an initial density of 0.5 × 10^6 ^cells in a humidified atmosphere (95% air, 5% CO2). The cells were harvested at 80 - 90% confluence by trypsin-EDTA (BioWhittaker) treatment, washed, centrifuged, the cell pellet resuspended in serum-free medium, and the cell number determined (P0 culture). For the *in vitro *experiment, the cells were use directly for subsequent passage 1 (P1) cultivation either in monolayer or embedded in the hydrogel. For the *in vivo *experiment the P0 cells were stored above liquid nitrogen until further use.

#### In vitro experiment

To compare the behavior of the disc cells in the hydrogels and in monolayer culture, 0.5 × 10^6 ^cells/500 μl hydrogel were cultured in one well of a 24-well plate. In parallel, 0.17 × 10^6 ^cells were seeded in 25 cm^2 ^cell culture flasks. Cultures were harvested at day 4, 7, and 14 and used for gene expression analysis.

#### In vivo experiment

The frozen cells from all six primary cultures were simultaneously re-activated prior to the experiment by seeding them once more into 75 cm^2 ^cell culture flasks at 2 × 10^6 ^cells/flask and expanding into confluence in the presence of 10% human serum and 1:1000 ITS liquid media supplement (insulin, transferrin, sodium selenite). They were harvested simultaneously by trypsinization (P1 cells). The harvested human cells were suspended in the implantation culture medium (consisting of a proprietary mixture of good manufacturing practice (GMP) grade phenol red - free culture medium, supplemented with 5% human serum), then mixed with the hydrogel solution (5 × 10^6^/ml gel solution) and transferred into the 2-mlcompartment of a dual chamber syringe. The second 0.5-ml-compartment was filled with SHPEG solution. The syringe was maintained at 4°C until use.

### Surgical implantation into and recovery from mice

Using the dual-chamber syringe, 1.25 × 10^6 ^IVD disc cells resuspended in 250 μl hydrogel were injected subcutaneously in the back of double-homozygous SCID hairless outbred model (Crl:SHO-Prkdc^scid^Hr^hr ^mice; Charles River Laboratories; Sulzberg, Germany). Polymerization of the hydrogel was achieved *in situ*. Two weeks later, the hydrogels were explanted, lysed for gene expression analysis or embedded in Tissue Tek, snap-frozen in liquid nitrogen and stored. As a control, hydrogels containing IVD cells from the same patients were cultured *in vitro *(250 μl in a 24-well) for two weeks (n = 6 patients).

### Histology

The frozen tissue samples were sliced by a cryotome to prepare 50 μm sections, and stained with haematoxylin-eosin (HE). The sections were analyzed by immunohistology with antibodies against collagen type I (Anti-human collagen type I 63170, lot 1467 K, MP Biomedicals; LLC, Solon, Ohio, USA), collagen type II (clone II-II6B3; 1 μg/ml; [25], and aggrecan (SM1353, Lot 040308, Acris Antibodies GmbH; Herford, Germany). The collagen type II antibody was obtained from the Developmental Studies Hybridoma Bank maintained by the Department of Pharmacology and Molecular Sciences, Johns Hopkins University School of Medicine, Baltimore, MD 21205, and the Department of Biological Sciences, University of Iowa, Iowa City, IA 52242, under contract NO1-HD-2-3144. Secondary antibodies were Cy3-conjugated IgG antibodies from Dianova (Hamburg, Germany). Control sections were prepared without primary antibody. The stained sections were analyzed by normal light or fluorescence microscopy (Axiophot, Zeiss; Jena, Germany). Photography was performed with an electronic camera (Axiocam, Zeiss).

### *In situ *hybridization

For the discrimination between cells of murine and human origin species specific *in situ *hybridization was done. Digoxigenin (DIG)-labeled mouse specific SINE/B1 and SINE/B2 and human specific Alu probes were generated as described previously [26]. Sections were washed three times in PBS containing 0.1% Tween at room temperature and then treated with 50 μg/ml proteinase K (Fermentas, Germany) in PBS containing 0.1% Tween for 10 min at 37°C. After washing in PBS, sections were treated with 0.25% acetic acid containing 0.1 M triethanolamine (pH 8.0) for 10 min. and then pre-hybridized for 1 h at 42°C in hybridization buffer containing 4 × saline-sodium citrate (SSC), 50% deionized formamide, 1 × Denhardts solution, 5% dextrane sulfate and 100 μg/ml salmon sperm DNA. Hybridization buffer was replaced by fresh buffer containing 0.2 ng/ml DIG-labeled probe (either human Alu or mouse SINE B1/B2) before target DNA and probe were denatured for 5 min. at 95°C. Hybridization was carried out for 16 h at 42°C in a wet chamber. Slides were washed twice in SSC for 5 min at RT and twice for 10 min. at 42°C in 0.1% SSC. Signals were detected using anti-DIG alkaline phosphatase conjugated Fab fragments (Roche, Germany) and nitroblue tetrazolium (NBT)/5-bromo-4-chloro-3-indolyl phosphate (BCIP) (Roche, Germany) as a substrate.

### Biochemistry

To analyze the extracellular matrix in the hydrogels the glycosaminoglycan (GAG) and collagen content was determined in the digested hydrogels and in the supernatants of the *in vitro *controls. Hydrogels were digested with 1 mg/ml papain (Sigma-Aldrich) in 0.1 M Naacetate, 0.01 M L-cysteine, 0.05 M Na^2^-EDTA, and 0.2 M NaCl (pH 6.0) at 60°C overnight. Culture supernatants were used without digestion. The GAG content was measured by the restrictive version of the dimethylmethyleneblue (DMB) assay, including guanidinium hydrochloride in the protocol [27] and using chondroitin-4-sulfate (Sigma-Aldrich) as a standard. Proteoglycan content was expressed as μg GAG per complete hydrogel (with a hydrogel volume of approx. 0.25 ml). Total collagen content was measured by the hydroxyproline assay based upon alkaline hydrolysis and reaction with chloramine-T and dimethylbenzamidine using gelatine as a standard [28]; the resulting values were expressed as μg collagen per complete hydrogel.

### Gene expression

To recover the cells for gene expression analysis from monolayers, an aliquot of the initial cell suspension after detachment of the cells was used and the cell pellet lysed in RNeasy lysis-buffer (Qiagen; Hilden, Germany). *In vitro *cultured hydrogels were digested with proteinase K (3 mg/ml; Sigma-Aldrich) for about 10 min. at 37°C, the digest was centrifuged and the cell pellet lysed in RNeasy lysis-buffer [24]. The gel pieces retrieved from the mice were snap-frozen in liquid nitrogen, ground in a mortar, then dissolved in RLT-buffer for RNA extraction. Total RNA was extracted using the RNeasy mini kit plus DNase I digestion according to the manufacturer's instructions (Qiagen). Complementary DNA (cDNA) was obtained by reverse transcription of total RNA using the Reverse Transcriptase Core kit (Eurogentec; Cologne, Germany) with EuroScript reverse transcriptase (Moloney Murine leukemia virus reverse transcriptase, 50 U/μl) and oligo-dT primers. Reverse transcription was performed in a total volume of 50 μl at 48°C for 30 min. in a thermocycler (Whatman Biometra; Goettingen, Germany).

Gene expression was analyzed by quantitative real-time PCR using an Applied Biosystems 7500 Fast Real-Time PCR System. The qPCR mastermix plus for SYBR green I (low ROX) kit from Eurogentec was used. Approximately 1 μg of cDNA, 100 - 300 mM primers and 2 × reaction buffer were pipetted to a total volume of 25 μl. Primers defining exon-intron boundary spanning segments were newly designed with the primer express 2.0 software (Applied Biosciences; Darmstadt, Germany), except primers for type II collagen (COL2A1) [29], and were obtained from BIOTEZ. Sequences of all primers used are summarized in Table 1. Glyceraldehyde-3-phosphate dehydrogenase (GAPDH) was used as a reference gene. The PCR reaction was started with an initial enzyme activation step at 95°C for 10 min (hot start), followed by 40 cycles of 95°C for 15 sec, 60°C for 30 sec, and 72°C for 30 sec. After the last cycle a dissociation curve was generated. Threshold cycles (Ct values) were determined using Sequence Detection System software (Applied Biosystems). Ct value of the reference gene was subtracted from the Ct value of the gene of interest (dCt) and the relative expression was expressed as 2^-dCt^. Median expression was calculated and is displayed in box plots relative to the median expression of the initial cell population. The boxes represent 25%/75% percentiles, mean (dotted line) and median (solid line) values.

**Table 1:** PCR primers

Gene	Forward/reverse	Product Length	Accession #
GAPDH^[a]^	AGAAAAACCTGCCAAATATGATGAC	126	NM_002046.3
			
	TGGGTGTCGCTGTTGAAGTC		

COL1A2^[a]^	GCTGGCAGCCAGTTTGAATATAAT	78	NM_000089.3
			
	CAGGCGCATGAAGGCAAGT		
			

COL2A1^[b]^	AGAGGTATAATGATAAGGATGTGTGGAAG	83	NM_001844.3
	GTCGTCGCAGAGGACAGTCC		

ACAN^[a]^	TGCATTCCACGAAGCTAACCTT	84	NM_013227
			
	GACGCCTCGCCTTCTTGAA		

HAS2^[a]^	GACCCGTTGGAGCAAGTCCTA	151	NM_005328.2
			
	ACCCCGGTAGAAGAGCTGGAT		

HAS3^[a]^	CGGTGGCTCAACCAGCAA	151	AF234839
			
	GTATAACCGTGGCAATGAGGAAGA		

SOX9^[a]^	ATCCAAGCGCATTACCCACT	132	NM_000346.3
			
	CGATTCTCCATCATCCTCCAC		

^a ^Primers designed in-house using Primer Express 2.0 software

^b ^[29]

### Statistics

Statistical analysis was performed using SigmaStat 3.0 (SPSS Science Software; Erkrath, Germany). Probability values p below 0.05 were considered to be significant. Specific details of the applied statistical functions are given in the figures respectively the corresponding figure legends. In general, both median and mean are documented, considering the typically high variability of human cell cultures.

## Results

The underlying philosophy of the experiments was to provide a continuous data set for the behavior of the intervertebral disc cells related to the hydrogel environment, as displayed during the cell expansion, after harvest into secondary culture as a monolayer or as an *in vitro *3D (hydrogel) culture, and finally isolated in the ectopic environment (within the hydrogel) of the subcutaneous location in the mouse.

### Rheological measurements

**Figure 1:**
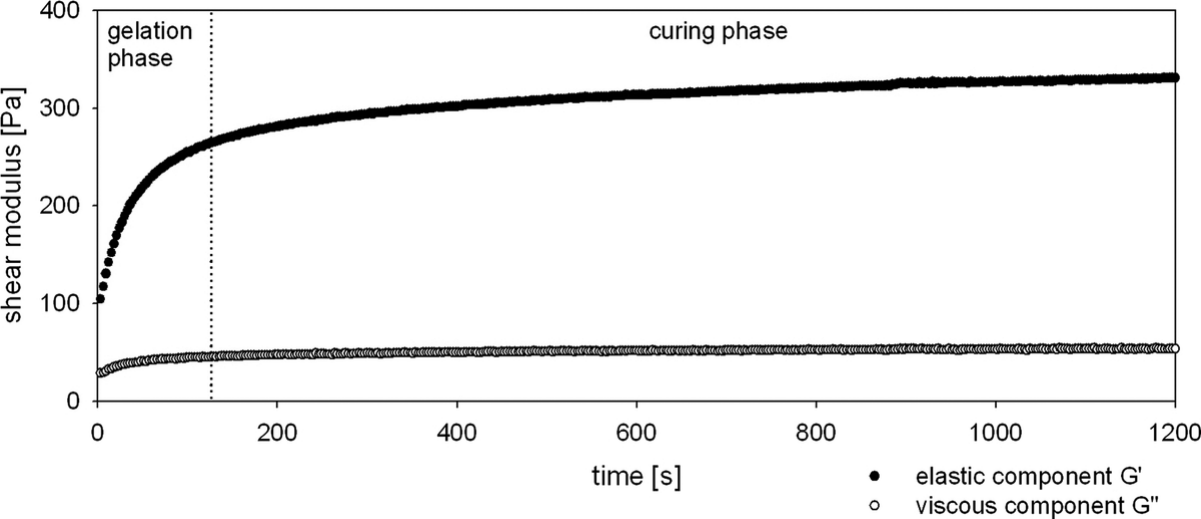
**Gelation process of the hydrogel**. The graph depicts the gelation process of the hydrogel separated into two phases, the gelation and the curing phase. The two gel components were mixed and injected onto the bottom plate of the rheometer using a dual-chamber applicator syringe. After adjusting the upper plate, the measurement started approximately 30 sec after gel mixture. The experimental conditions of the gel formation were: temperature 37°C, shear strain 0.05%, shear stress 1 Pa, oscillation frequency 1 Hz. Storage modulus G', representing the elastic component of the gel, and loss modulus G", representing the viscous component of the gel, were calculated every third second. The gelation is completed after approximately 2-3 minutes; thereafter the visco-elastic properties of the hydrogel are constant.

Rheological measurements were performed with nascent gel formulations produced out of the dual chamber syringe as described previously [24]. One measurement is exemplified in Figure 1. The gelation process of the gel is completed after approximately 2 - 3 minutes; thereafter the visco-elastic properties of the hydrogel remain nearly constant.

A total of 24 finished products were analyzed (Table 2). Thirteen measurements were performed with freshly prepared hydrogel components, five measurements were performed after storage gel components at 4°C for 24 h and six measurements were performed after storing the gel components at 4°C for 72 h. The hydrogel production process could be successfully standardized; thus hydrogels with reproducible visco-elastic properties can be produced. There was no significant difference in storage modulus G' (measurements 1-3) and loss modulus G" (measurements 4-6) between the three time points (Table 2). Initial storage of the liquid gel precursors for 72 h does not alter the visco-elastic properties of the polymerized hydrogel. This storage time was chosen to simulate the necessary transportation of the hydrogel components to the user after preparation of the gel solution/cells mix.

**Table 2:** Effect of pre-gelation storage time at 4°C on the visco-elastic properties of the hydrogel

storage time	0 h	24 h	72 h
measurement #	1	2	3

number of hydrogels	13	5	6

mean G'[Pa]	265.2	377.8	187.7

standard deviation	158.1	78.1	156.4

measurement #	4	5	6

number of hydrogels	13	5	6

mean G''[Pa]	39.7	28.9	25.0

standard deviation	22.6	10.3	9.8

Storage modules G' and loss modules G" were determined from hydrogels produced immediately after preparing the gel components (0 h), and after storing the liquid gel components for 24 h or 72 h at 4°C. Mean value comparison using ANOVA showed no difference of storage modules between measurements # 1 - 3 and no difference of loss modules between measurements # 4 - 6 (*P *< 0.05)

### *In vitro *reference culture

**Figure 2:**
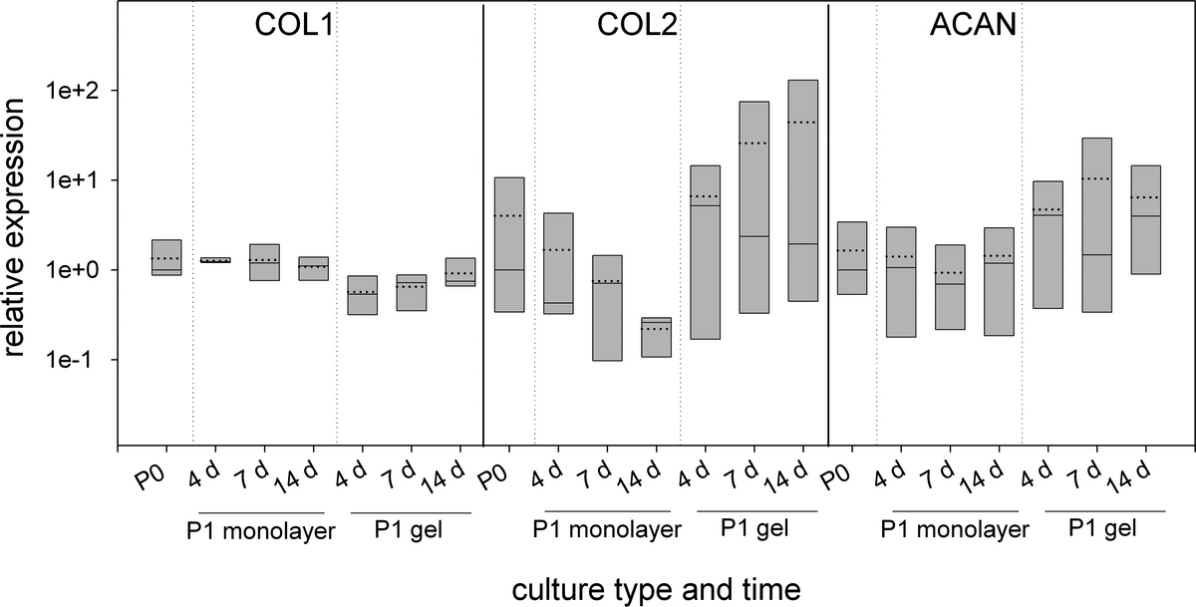
**mRNA expression profiles of human disc cell preparations cultured *in vitro *in monolayer or hydrogel**. P1 cells were harvested at days 4, 7, and 14 and the expression of collagen type I (COL1), collagen type II (COL2), and aggrecan (ACAN) was analyzed. Expression values of the P1 cultures are expressed relative to the median expression of the cells at the end of the expansion phase (P0) (median because the data are not normally distributed). Boxes represent 25%/75% percentiles, mean values (dotted line) and median values (solid line).

In an *in vitro *reference culture the alterations in gene expression of the matrix genes collagen type I, type II and aggrecan were analyzed at different time points during the culture of disc cells in monolayer and hydrogel cultures. This data set expression was compared to the expression data of the initial cell population (P0) used for the subculture (Figure 2). Collagen type I expression was almost unchanged over time and independent from culture type. Collagen type II expression decreased in monolayer cultures, at the end of the 14 days monolayer culture the median expression was 4-fold less (mean 18-fold) than the expression in the P0 cells. In contrast, in hydrogels the expression at all three time points was higher than in the P0 cells (median: 2 - 5-fold; mean: 1,7 - 11-fold). Although these differences were not statistically significant due to restricted number of patients (n = 4) and high individual variations a clear tendency was visible: the hydrogel culture improved the collagen type II expression compared to the monolayer (median: 10-fold, mean: 200-fold higher at day 14). Aggrecan expression was less effected by culture type. Nonetheless the same tendency for improved expression in hydrogels can be seen as for collagen type II mRNA expression.

### Mouse subcutaneous implants and *in vitro *cultured hydrogels

The subcutaneous implantation into mice was intended to extend the *in vitro *data towards an *in vivo *application. In preliminary experiments, we found that gels without cells were completely resorbed within 8-12 weeks post implantation in SCID mice (data not shown). In order to achieve rapid accumulation of extracellular matrix, we decided to inoculate the hydrogels with a relatively high cell density. At the same time, this high density would give us pharmacological information on overdosing effects concerning the viability of cells in the gel under the presumed restrictive nutritional environment within the subcutis as compared to the unlimited supply in culture medium. The entire *in vivo *experiment was performed with cryo-conserved, in secondary culture expanded disc cells from six different patients, similar as planned to the future therapeutical use.

**Figure 3:**
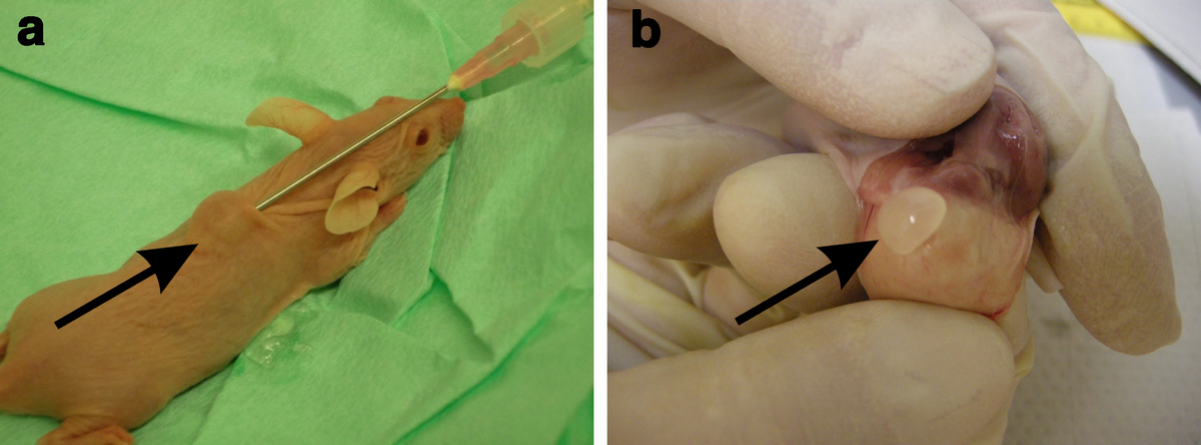
**Injection and harvest of cell-loaded hydrogels**. **A**: Injection from dual syringe and 1.3 mm Ø needle. The hydrogel (arrow) polymerizes upon injection and forms a stable subcutaneous protrusion. **B**: Hydrogel (arrow) at harvest. Note the absence of vascular and connective tissue ingrowth.

Upon injection, the gel polymerized instantly and produced approximately 0.25-ml globules that remained stable and visible during the entire 14 days of incubation in the animal (Figure 3A). For comparison, cell-laden hydrogels were maintained *in vitro *in culture for the same time period.

At harvest, the implants presented as a separate entity within the animals. The opaque gels could be peeled out of the subcutis without any damage (Figure 3B). In contrast to their initial plasticity, the gels were solid to the grip and displayed some elasticity and structural stability.

**Figure 4:**
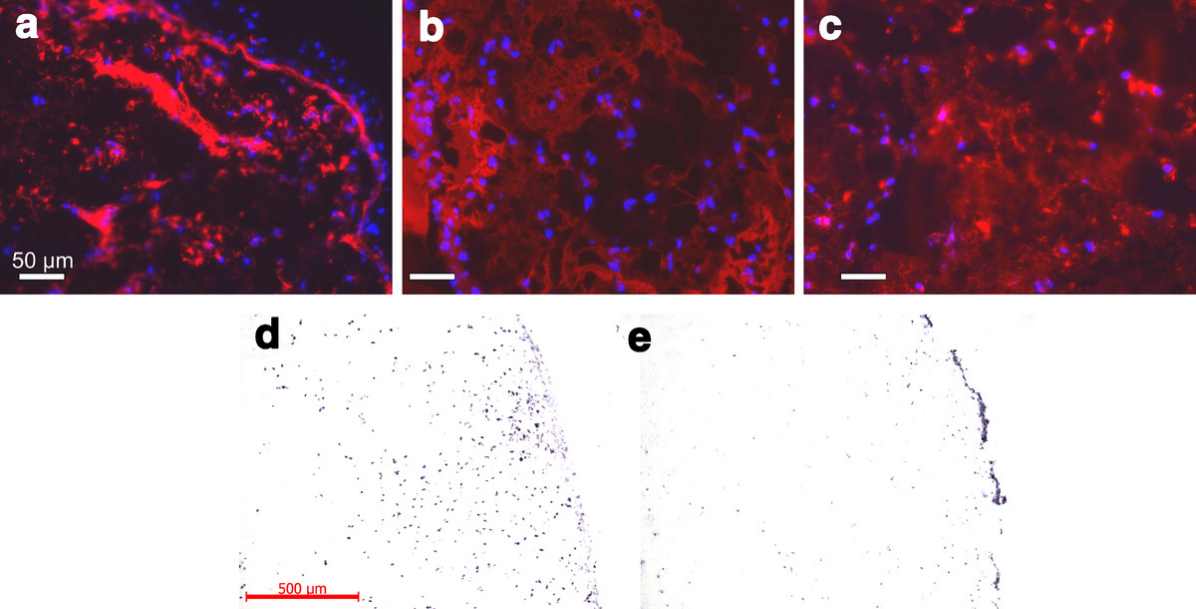
**Immunohistochemistry staining and species specific genomic *in situ *hybridization of an implanted hydrogel upon harvest**. **A**: Detection of collagen type I; **B**: collagen type II; **C**: aggrecan by immunohistochemistry. The blue dots are the DAPI stained nuclei. Note the even distribution of the nuclei across the samples, except for the edge in A. Species specific *in situ *hybridization discriminates between cells of human origin (**D**) and cells of murine origin (**E**). Mouse cells are predominantly found at the edge of the xenotransplant, human cells are distributed evenly within the transplant.

Concerning the hydrogel itself, histological inspection revealed a rather homogenous distribution of the disc cells within the hydrogel (Figure 4A-C). There were no obvious regions with particular features such as higher densities of viable cells at the edge or the centre. To verify the presence and the distribution of human cells in the transplants a species specific genomic hybridization was performed. It was possible to discriminate between cells of human origin and cells of murine origin. Mouse cells are predominantly found at the edge of the xenotransplant (Figure 4E) (corresponding with the collagen type I staining); human cells are distributed evenly within the transplant (Figure 4D), confirming the above notion of the initial rather homogenous cell distribution. It appears that few cells with positive mouse Alu hybridization are also present within the hydrogel (Figure 4E). These might be false positive signals, as the probes to mouse Alu sequences tend to weakly bind to human Alu sequences. Since there are fewer mouse sequences than human Alu, the detection reaction with NBT/BCIP has to be intensified in order to visualize bound probes per nucleus in mouse cells. In this case, the unspecific binding on human cells may also appear and cause the present false positive signals on some cells.

The inner portions of the gel were positive for collagen type II and aggrecan immunostaining with the stain being either evenly distributed or clustered; collagen type I was found at the edges, and to a much lesser degree, in the centre of the gels. The accumulation of collagen type I at the edges of the *in vivo *transplants correlates with the appearance of a fibroblastic cell ring at the border of the gels, which could be seen in the *in vitro *cultured gels (data not shown).

**Figure 5:**
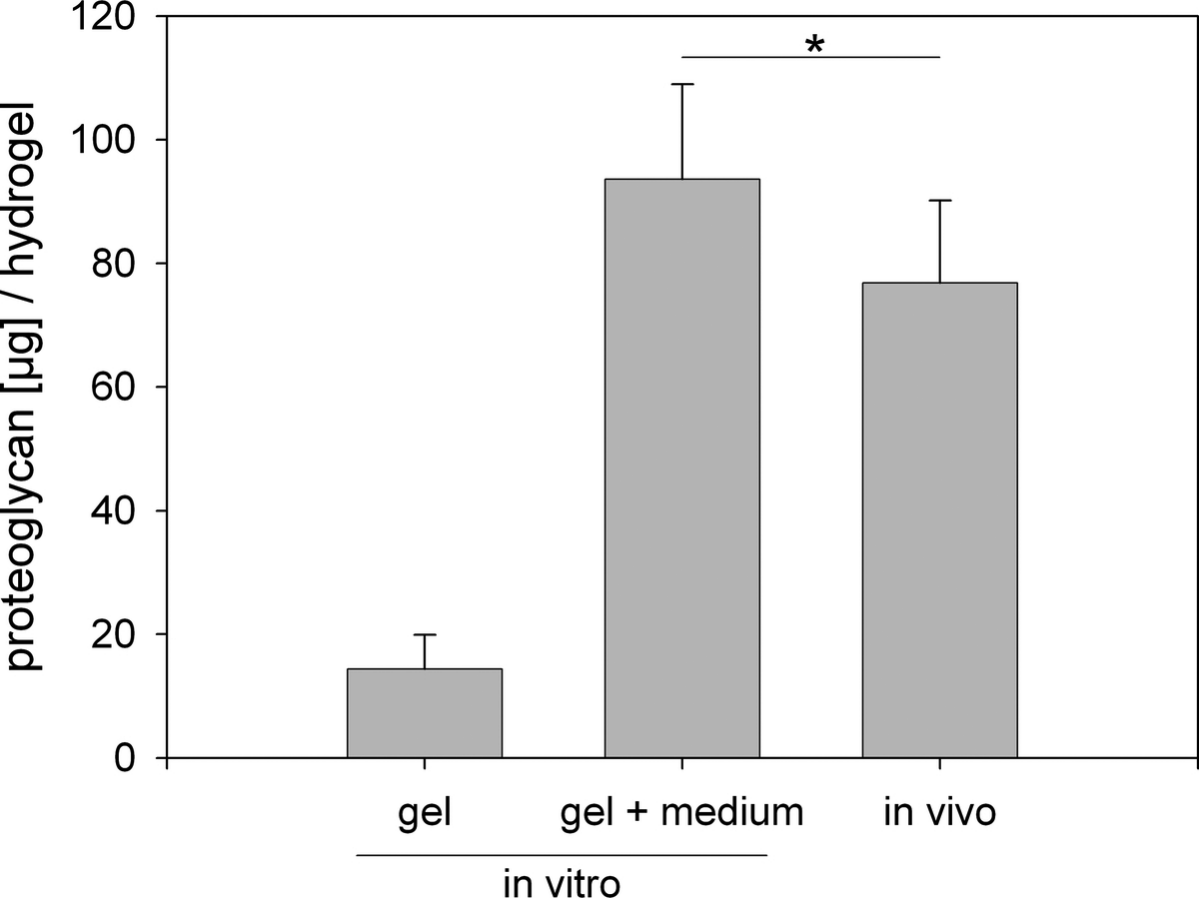
**Glycosaminoglycan production from cells of *in vitro *maintained and of *in vivo *implanted hydrogels**. Note that most of the glycosaminoglycans (GAG) from the *in vitro *cultures were found in the culture medium (middle bar). Mean values (n = 6) and standard deviation are shown. *In vitro *gel: GAG content of the *in vitro *cultured hydrogel; *in vitro *gel + medium: GAG content of the *in vitro *cultured hydrogel plus the GAG released into the combined medium supernatant from two weeks of culture; *in vivo*: GAG content of the *in vivo *implanted hydrogel. Statistic: paired *t*-test comparing the *in vitro *group (gel + medium) and the *in vivo *group, * = *p *< 0.05.

**Figure 6:**
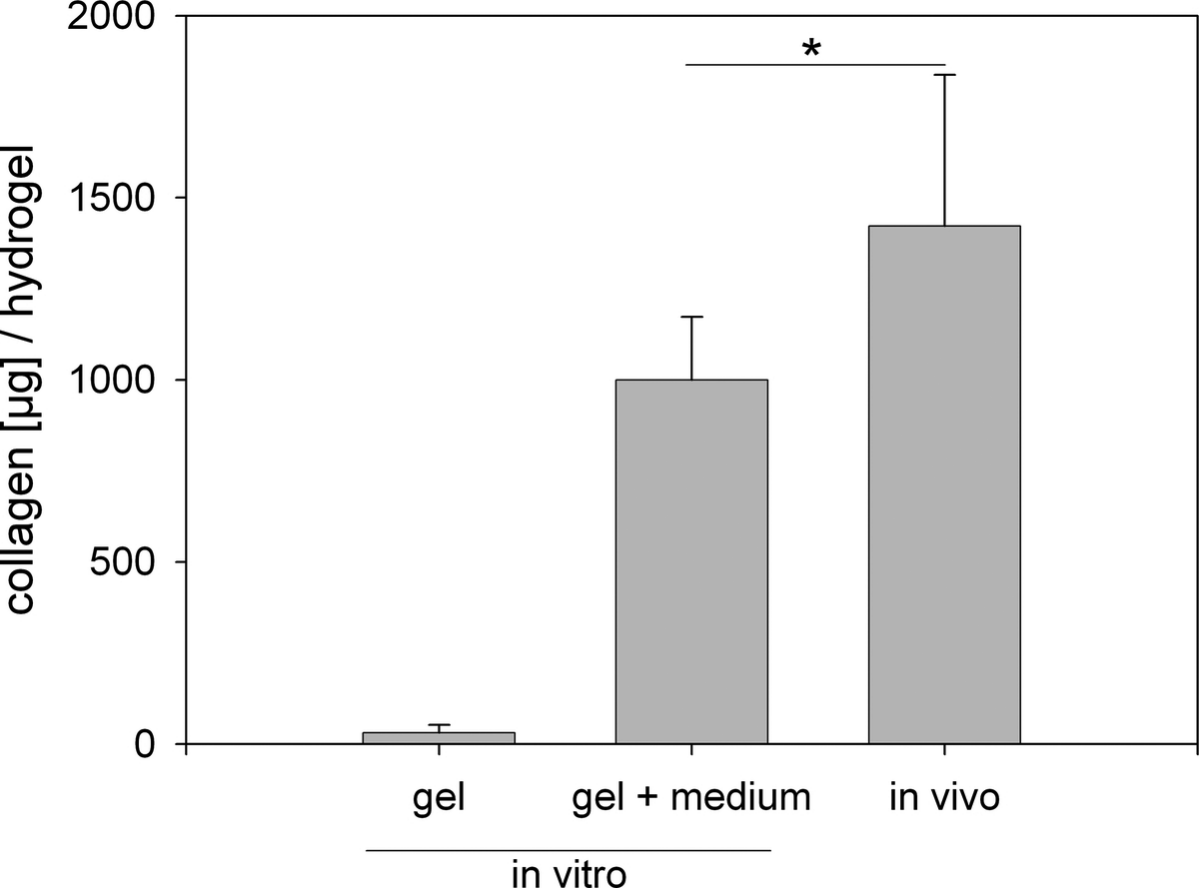
**Cumulated collagen production from cells of *in vitro *maintained and of *in vivo *implanted hydrogels**. Note that most of the collagens from the *in vitro *cultures were found in the culture medium (middle bar). Mean values (n = 6) and standard deviations are shown. *In vitro *gel: collagen content of the *in vitro *cultured hydrogel; *in vitro *gel + medium: collagen content of the *in vitro *cultured hydrogel plus the collagen released into the combined medium supernatant from two weeks of culture; *in vivo*: collagen content of the *in vivo *implanted hydrogel. Statistic: paired *t*-test comparing the *in vitro *group (gel + medium) and the *in vivo *group, * = *p *< 0.05.

In contrast to these staining patterns from the subcutaneous hydrogels, the *in vitro *cultured hydrogels displayed only very poor immunostaining for any antigen (data not shown, see also biochemical analysis in Figures 5 and 6). Upon macroscopic inspection, the hydrogels maintained in tissue culture for 14 days remained clear and soft with high plasticity, quite in contrast to the gels harvested from the mice. The results of the bulk biochemical analysis may explain the contrasting findings. Cultured hydrogels contained more than five times less glycosaminoglycans (GAG) than implanted gels (Figure 5). The collagen deposition in implanted gels was even 50-fold higher as in cultured gels (Figure 6). On the other hand, when the GAG and collagen content of the culture medium was taken into consideration, the total GAG and collagen output of the cultured cells was seemingly rather equal to that of the implanted cells (Figure 5 und6). *In vitro *cultured cells produced slightly but significantly more GAG than the subcutaneous cultured cells (1.2-fold), conversely collagen accumulation was significantly higher in *in vivo *hydrogels (1.4-fold). Taken together, the entire process of matrix deposition *in vivo *could be considered to be more efficient towards formation of a functional tissue whereas *in vitro *conditions seemingly delayed that process.

**Figure 7:**
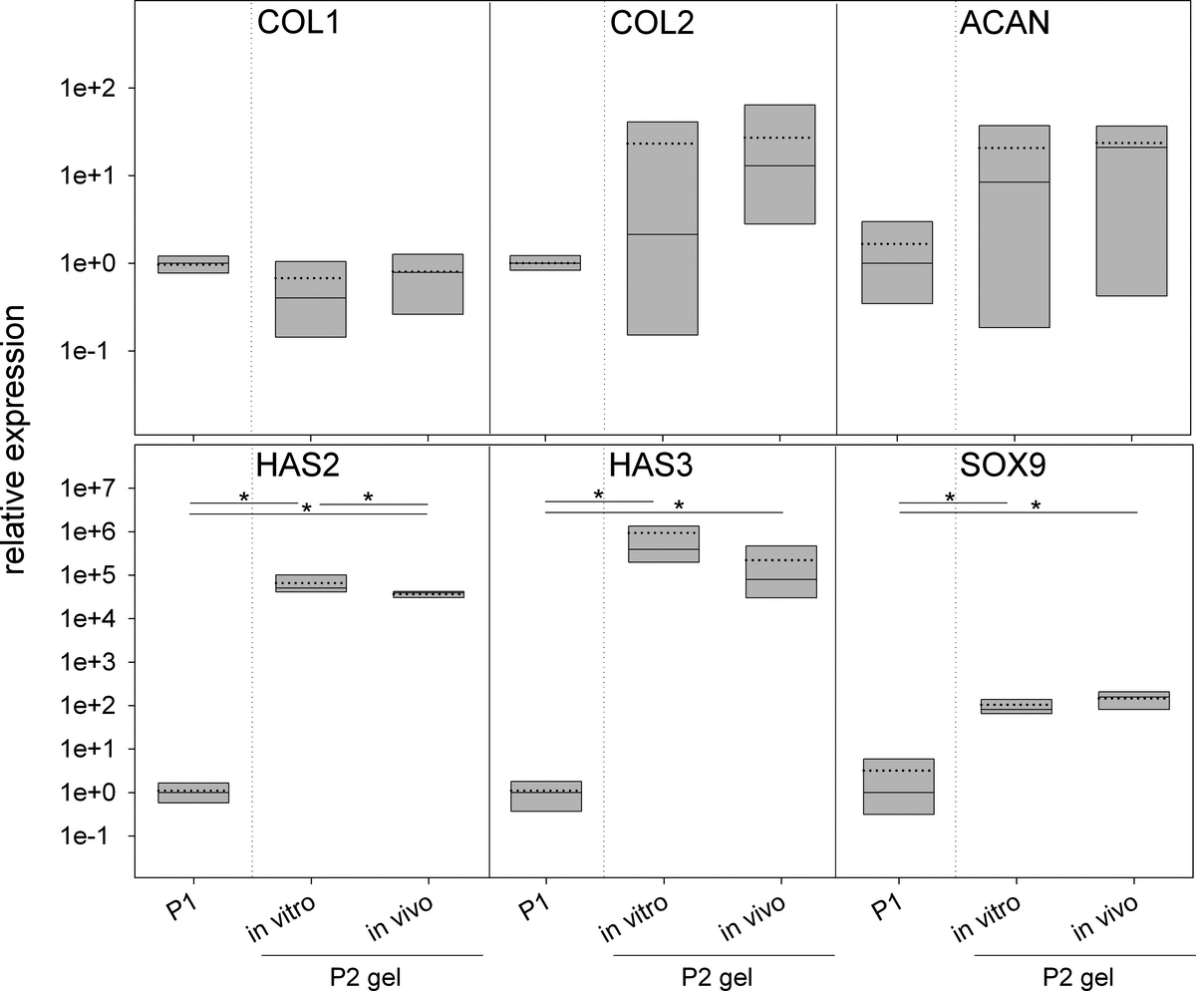
**mRNA expression profiles of the human disc cells *in vitro *and *in vivo***. The expression of collagen type I (COL1), collagen type II (COL2), aggrecan (ACAN), hyaluronan-synthase-2 (HAS2), hyaluronan-synthase-3 (HAS3) and SRY (sex determining region Y)-box 9 (SOX9) was analyzed in P2 cells hydrogels cultured for two weeks *in vitro *or implanted in mice for two weeks and compared to the median expression of the P1 cells (median because not all data were normally distributed). Statistic: One Way repeated Measures Analysis of Variance was performed if the data of all groups were normally distributed, if not, Friedman Repeated Measures Analysis of Variance on Ranks was done. * = *p *< 0.05. Boxes represent 25%/75% percentiles, mean values (dotted line) and median values (solid line).

At time of harvest, the deposition of ECM within the hydrogels was accompanied by a still active gene expression in the human cells, analyzed by RT-PCR for a number cartilage- and disc-specific factors including collagen types I, II, aggrecan, hyaluronan synthases-2 and -3 (HAS2, HAS3), and SOX9 as a chondrocytic/disc cells key transcription factor (Figure 7). The median expression was compared to the expression levels of the initial cell population (P1) used for the production of the cell/hydrogel constructs (P2). As seen in the *in vitro *experiment, no significant changes in the transcription of genes coding for the matrix molecules collagen type I (COL1), type II (COL2), and aggrecan (ACAN) could be detected. Although both *in vitro *and *in vivo *hydrogels showed by trend higher expression levels for COL2 and ACAN, this effect was not statistically significant. Hydrogel culture had an extensive effect of the transcription of the hyaluronan-synthase genes and of the transcription factor SOX9. HAS2 and HAS3 expression was up-regulated to 4-5 orders of magnitudes in hydrogels cultures compared to the initial P1 cell population. Expression of both genes was slightly higher in *in vitro *hydrogels than in *in vivo *hydrogels (1.2-fold for HAS2 and 5-fold for HAS3), an effect being statistically significant for HAS2. Compared to P1 cells mRNA for SOX9 was found 80 times higher in *in vitro *hydrogels and about 160 times higher in *in vivo *transplants. As SOX9 expression accompanies chondrocytes and disc cells differentiation and cartilage/disc specific matrix production, the higher expression in hydrogels refers to a more differentiated phenotype compared the P1 cells.

## Discussion

The present study generated data that support the idea of disc repair through the administration of intervertebral disc cells harvested from a disc prolaps and captured within an injectable hydrogel. *In vitro *and *in vivo *data point to a sustainable functionality of phenotypic disc cells within the hydrogel. Even though the present study did not investigate cell viability in particular, the gene expression data indicate high viability in the present settings. Earlier work provided evidence that the viability of a number of cell types embedded into the hydrogel, including disc cells, is greater than 90% [24]. In particular the rapid deposition of cartilagenous ECM and the significant levels of hyaluronan synthase isozymes-2 and -3, and SOX9 suggest cell functionalities towards those needed for the support of damaged intervertebral disc cores (the original nucleus pulposus). The reproducibility of the particular visco-elastic properties (rheological data) encourages administration of the gel mixture within the confinements of the damaged disc. The biomechanical properties of the hydrogel in intervertebral discs remain to be determined in specific studies to follow and have been in part presented by Beger [30].

Hydrogels are rapidly gaining attention as highly versatile carriers of bioactive compounds and cells in tissue engineering approaches [31-33]. We decided on a hydrogel formulation that is governed by aspects of drug safety and clinically established subcomponents. Contrary to many approaches, we refrained from choosing a base compound (such as collagen), that in itself may exert cell regulative properties towards chondrocyte-like cell types as they are found in intervertebral discs. This decision is based, among other considerations, on the well-known fact that chondrocytes and nucleus pulposus cells differentiate best in agarose, a compound that exerts no metabolic effect towards the cells and possesses a maximum of retentive properties towards newly synthesized ECM components, keeping the ECM in the vicinity of the cells [34,35]. Alginate is similar to agarose, in this aspect [36,37]. Serum albumin can be purchased as prescription drug. The chosen cross-linker, polyethylene glycol, has a long-standing history as a pharmaceutical additive and, as plasma expander, a proven history of non-toxicity. Albumin is readily degraded *in vivo*, with no toxic or otherwise critical intermediates or end products being generated in this process. Furthermore, in contrast to ester-based biopolymers, degradation of albumin does not set free acidic components. Thus a shift to lower pH is not expected during degradation of the hydrogel. The chemical polymerization reactions, starting from the maleimide activation of albumin and ending in the dual chamber application modality, possess no pharmaceutical problems, neither.

The option to deliver, via an appropriate injection device, several components simultaneously, the therapeutic combination of cells, scaffold and regeneration-promoting substances (such as the hyaluronan applied here) was demonstrated in the presented animal experiment. No animal displayed any sign of distress, and recovery from anesthesia was speedy because of the short time needed for the injection. The injected mixture polymerized *in situ *and did not dissipate upon injection. There was a distinct border between the host tissue and the implant that could not be surmounted by invasive cell types of the animals' subcutis, just as published recently [5]. A subsequent histological examination of the *in vivo *maintained hydrogels revealed that the hydrogel prevented tissue ingrowth in part by its anti-angiogenic properties. This effect was then experimentally explored in various test systems and has been reported elsewhere [5].

One surprising key finding of these experiments was the presence of a balanced primary GAG and collagen biosynthesis by the cells for the cultured hydrogel as well as for the implanted hydrogels even though under the *in vitro *conditions, there was much less deposition of GAG and collagen within the gel structure. Regarding mRNA expression among the three groups of cells, P1cells, P2 *in vitro *hydrogel, and P2 *in vivo *hydrogel, there was a difference in favor of higher transcriptional activities in the hydrogels, but especially the difference between in *in vitro *and *in vivo *hydrogels resulted in only marginal differences from a functional standpoint in that both conditions led to approximately identical protein output by the cells. Baseline metabolic conditions may not be responsible for that effect since GAPDH expression levels were quite similar under all three conditions at time of harvest (data not shown). What differed, however, was the effective deposition of the produced ECM molecules within the hydrogels *in vitro *and *in vivo*.

Thus, the resulting difference in matrix deposition may have not been due to overall metabolic conditions but rather related to specific effects, such as differential matrix metalloprotease activities *in vivo *versus *in vitro *or the encapsulation by murine tissue. The higher deposition of GAG and collagen molecules in implanted gels may also arise from biomechanical stimulation of the construct in the *in vivo *situation. Additionally, the effect may be facilitated by the hypoxic conditions in a non-vascularised subcutaneous implant in mice [38,39]. The differential deposition of collagen type I and II within the *in vivo *incubated gels may represent another hint for specific processing of such matrix molecules or of spontaneous differential evolvement of cell types (chondrocytes/nucleus pulposus cells and fibrochondrocytes). These speculations need further investigations, though. The additional high levels of SOX9 message also differentiate cells from unspecific connective tissue cells and underscore their chondrogenic roots.

The pronounced expression of hyaluronan synthases supports the function of the hydrogel/cell mixture as disc regenerating element. HAS2 is the main producer of hyaluronan in chondrocytes, has been reported to be highly expressed in the intact nucleus pulposus [40,41]. HA is known to significantly contribute to osmotic pressure by locally binding large molar quantities of water [42]. The effect is enlarged by albumin, as in synovial fluid [43]. It has been investigated in many physiological contexts, including for example kidney, where HA supports water retentive activity [44], abdominal wall musculature, where HA supports the maintenance of the interstitial pressure [45], and in growth plates, where it facilitates in volume expansion of the hypertrophic cells [46,47], to name some functions. HAS activity may therefore be chiefly responsible for the maintenance of the high hydrostatic pressure of a healthy disc.

## Conclusions

The present study provides experimental evidence to support the idea of disc repair by intervertebral disc cells harvested from a disc prolaps and applied together with an injectable hydrogel. *In vitro *and *in vivo *data suggest that human disc cells even after a cryopreservation step are maintained in a functionally mature stage for at least two weeks when maintained in the hydrogel. Moreover, the molecular data derived from cells in hydrogel *in vitro *and *in vivo *corroborate the hypothesis that *in vitro *cultivation of cells in the hydrogel has some relevance for the development of such implants for clinical applications as well. In particular the rapid deposition of cartilagenous ECM and the significant levels of hyaluronan synthase isozymes-2 and -3 of the propagated disc cells suggest functionalities needed for the support of damaged intervertebral discs. In continuation of these experiments, a large animal disc injury model for intervention in sheep based on hydrogel/cell composites application has already been completed. The data are currently evaluated, with early assessment indicating that upon further refinement the present approach will probably become suitable for disc repair in the near future.

## Competing interests

KB and JAM have applied for a patent related to the use of the hydrogel material. CG holds shares in the TETEC AG.

## Authors' contributions

KB conception and design of the study, analysis and interpretation of data, statistical analysis, drafting manuscript. CS carried out cell culture experiments, gene expression analysis, and histology. CO carried out biochemical assays. CG design and coordination of the study, revision of the manuscript. NL performed rheological measurements. AB selection of human donors, ethic approval for the use of human tissues. ES performed species specific *in situ *hybridization. WKA animal study, animal experiment approval, revision of the manuscript. JAM performed animal experiments, analysis and interpretation of data, drafting and revision of the manuscript. All authors read and approved the final manuscript.

## Pre-publication history

The pre-publication history for this paper can be accessed here:

http://www.biomedcentral.com/1471-2474/13/54/prepub
